# Ribosomal protein and biogenesis factors affect multiple steps during movement of the *Saccharomyces cerevisiae* Ty1 retrotransposon

**DOI:** 10.1186/s13100-015-0053-5

**Published:** 2015-12-08

**Authors:** Susmitha Suresh, Hyo Won Ahn, Kartikeya Joshi, Arun Dakshinamurthy, Arun Kananganat, David J. Garfinkel, Philip J. Farabaugh

**Affiliations:** Department of Biological Sciences and Program in Molecular and Cell Biology, University of Maryland Baltimore County, Baltimore, MD 21250 USA; Department of Biochemistry & Molecular Biology, University of Georgia, Athens, GA 30602 USA; Present address: Division of Infectious Diseases, Department of Internal Medicine, Stanford University School of Medicine, Stanford, California 94305 USA; Present address: Department of Nanosciences and Technology, Karunya University, Karunya Nagar, Coimbatore, 641 114 Tamil Nadu India

**Keywords:** Retrotransposition, Host factors, Programmed frameshifting, Ribosomal protein insufficiency, Ribosome biogenesis

## Abstract

**Background:**

A large number of *Saccharomyces cerevisiae* cellular factors modulate the movement of the retrovirus-like transposon Ty1. Surprisingly, a significant number of chromosomal genes required for Ty1 transposition encode components of the translational machinery, including ribosomal proteins, ribosomal biogenesis factors, protein trafficking proteins and protein or RNA modification enzymes.

**Results:**

To assess the mechanistic connection between Ty1 mobility and the translation machinery, we have determined the effect of these mutations on ribosome biogenesis and Ty1 transcriptional and post-transcriptional regulation. Lack of genes encoding ribosomal proteins or ribosome assembly factors causes reduced accumulation of the ribosomal subunit with which they are associated. In addition, these mutations cause decreased Ty1 + 1 programmed translational frameshifting, and reduced Gag protein accumulation despite at least normal levels of Ty1 mRNA. Several ribosome subunit mutations increase the level of both an internally initiated Ty1 transcript and its encoded truncated Gag-p22 protein, which inhibits transposition.

**Conclusions:**

Together, our results suggest that this large class of cellular genes modulate Ty1 transposition through multiple pathways. The effects are largely post-transcriptional acting at a variety of levels that may include translation initiation, protein stability and subcellular protein localization.

**Electronic supplementary material:**

The online version of this article (doi:10.1186/s13100-015-0053-5) contains supplementary material, which is available to authorized users.

## Background

The *Saccharomyces cerevisiae* Ty (**T**ransposons of **y**east) retrotransposons are members of the LTR (long terminal repeat) group and are similar to retroviruses both structurally and functionally [1, 2]. Like retroviruses, Ty elements undergo reverse transcription that occurs within virus-like particles (VLPs) formed from structural and enzymatic proteins encoded by two genes, *GAG* and *POL*. Ty elements are valuable as models for human retroviruses; several groups have exploited yeast genetic tools to identify genes encoding Ty host factors that modulate transposition. Knowing how these factors affect Ty retrotransposition can provide clues as to what host processes affect retrovirus or retrotransposon replication and pathogenicity. Genome-wide forward genetic screens identified host factors that are required for (cofactor genes) or prevent (restriction genes) retrotransposition by Ty1 [3–7]. The most salient feature of the genes identified in these screens is the diversity of function of their encoded products, including roles in transcription, chromatin structure and modification, intracellular signaling, cytoplasmic protein synthesis, DNA repair, RNA processing and cell cycle regulation among others. Among the most statistically overrepresented host cofactor genes are those encoding cytoplasmic ribosomal proteins [7] suggesting that Ty transposition might depend on efficient biogenesis of ribosomes. Host factors for other plus stranded viruses in yeast have not been found to be as diverse. Prominent among these is the endogenous L-A virus of *S. cerevisiae*. It supports the replication of satellite dsRNA molecules, one of which encodes a peptide toxin lethal to uninfected cells [8]. Maintenance of L-A and the satellites depends on availability of the large (60S) ribosomal subunit [9], implying a more global role of protein synthesis for positive stranded viruses. Because, unlike Ty1, L-A has no integrated DNA form, it does not share a dependence on genes such as those involved in transcription, chromatin recombination and DNA repair. Its dependence on 60S abundance may relate to the L-A mRNA not being polyadenylated since polyA tails facilitate 60S joining during translation initiation [10]. Thus, reduced 60S availability could reduce L-A mRNA translation relative to bulk poly(A)^+^ mRNA (reviewed in [11]). Ty1 expresses an abundant, poly(A)^+^ mRNA and depends on both 40S and 60S availability so its dependence on the translation machinery may have a different origin. Also, only three Ty1 cofactor genes were also identified as L-A host factors—*SKI1*/*KEM1/XRNI*, *SKI2* and *SKI8*—and their Ty1 phenotype is opposite to their effect of L-A virus; these factors are required for Ty1 mobility but restrict L-A propagation. Therefore, Ty1 and L-A occupy distinct genetic niches with respect to their dependence on host proteins.

Ty elements, as well as many viruses and virus-like elements including L-A, employ an unusual translational control mechanism—programmed translational frameshifting [12]. The Ty and L-A frameshift mechanisms are distinct. Ty elements employ +1 frameshifting, in which translation shifts one base in the downstream or 3′ direction, while L-A uses -1 frameshifting, shifting one base in the opposite direction. The Ty1-encoded enzymatic (Pol) protein is encoded as a fusion to the upstream-encoded Gag structural protein by +1 frameshifting at a 7 nt RNA signal [13]. A similar or identical signal is used in all but the Ty5 element. The frequency of Ty1 frameshifting is approximately 40 % measured in a reporter gene construct containing only the frameshift signal [13]. In the intact Ty1 element the Gag-Pol protein is expressed at 3 % the amount of the Gag protein, suggesting a further ~10-fold reduction in expression of Gag-Pol, which may result from either a translational effect during elongation through the *POL* gene or reduced stability of Gag-Pol relative to Gag protein; changes to this ratio blocked retrotransposition [14]. Altered Gag to Gag-Pol stoichiometry also reduces transposition of many other viruses [15–20]. Because retrotransposition frequency requires a specific level of programmed frameshifting, that process could explain the dependence of retrotransposition on efficient ribosome biogenesis.

In addition to cellular cofactor and restriction genes that affect Ty1 transposition, a protein expressed from subgenomic internally initiated Ty1i transcripts (Gag-p22) containing the C-terminal half of Gag is a self-encoded restriction factorthat inhibits transposition and controls Ty1 copy number [21]. Gag-p22 antagonizes VLP function by interfering with assembly of VLPs and assembly foci [22], called T-bodies [23] or retrosomes [24]. Well-known Ty1 cofactors such as *SPT3* and *XRN1*, which are implicated in full-length transcription [25], and RNA turnover and VLP function [26–28], respectively, influence the level of Ty1i RNA [21]. However, additional cellular genes that modulate Ty1i/Gag-p22 expression remain to be discovered, and in fact, may be present in Ty1 cofactor or restriction gene collections. A clue to what types of factors might influence this effect is the fact that formation of retrosomes requires co-translational insertion of the Ty1 Gag protein into the endoplasmic reticulum (ER) [22]. Interfering with ER insertion blocks formation of retrosomes and the Gag protein produced is more rapidly degraded. This suggests that some Ty1 cofactor genes might encode factors required for Gag ER insertion.

To gain a more thorough understanding of the relationship between ribosome biogenesis and Ty1 transposition, we analyzed the effect on Ty1 transposition of chromosomal deletions that remove structural proteins of the 40S and 60S ribosomal subunits as well as proteins involved in ribosomal processing or protein synthesis. We show that translation-associated cofactor deletion mutants affect Ty1 transposition through a combination of mechanisms. Most of the mutants tested show reduced accumulation of the corresponding ribosomal subunit, significantly decreased +1 programmed translational frameshifting at the Ty1 site, and reduced expression of Gag protein despite expressing at least normal amounts of Ty1 mRNA. Interestingly, several ribosome subunit mutants also express more Ty1i RNA relative to Ty1 mRNA and significant amounts of Gag-p22 and its C-terminally processed product, Gag-p18, consistent with the idea that producing more of the transpositional inhibitor Gag-p22 contributes to the Ty1 defects in these mutants [21]. Together, our results suggest that multiple post-transcriptional processes are required for optimal Ty1 transposition.

## Methods

### Media and yeast strains

Yeast genetic techniques and media were used as described previously [29, 30]. Strains from the haploid *MAT*α deletion collection [31] were obtained from Invitrogen (Carlsbad, CA). The mutant strains, constructed in BY4742 (*MAT*α *his3-*∆*1 leu2-*∆*0 lys2-*∆*0 ura3-*∆*0*) [32] were transformed with pJC573, a *URA3*-based integrating plasmid carrying an active Ty1 element tagged with a modified indicator gene *his3-AI*, which cannot recombine with the *his3-∆1* allele present in BY4742 to generate a functional *HIS3* gene [5]. The centromere-based Ty1 overexpression plasmid pGTy1*his3-AI* [21] was also introduced into BY4742 and an isogenic *rpl1B*Δ mutant.

### Frequency of Ty1*his3-AI* mobility

Mobility of Ty elements in each mutant strain was determined essentially as described [5, 33]. Strains were streaked for single colonies on SC –Ura plates at 20 °C and a single colony suspended in SC –Ura liquid and ~10^3^ cells inoculated into each of six tubes and incubated at 20 °C to saturation. Aliquots were plated on SC –Ura and SC –His –Ura and incubated at 30 °C. The frequency of Ty1*his3-AI* was calculated by dividing the average number of His^+^ Ura^+^ cells per milliliter by the average number of Ura^+^ cells per milliliter. Mobility of cells expressing a *GAL1*-promoted Ty1*his3-AI* plasmid (pGTy1*his3-AI*) was determined as described by Saha et al. [21].

### Ty1 frameshifting efficiency

Ty1 programmed +1 frameshifting efficiency was measured as described [13]. Briefly, the assay employs two reporter plasmids that include a translational fusion of the first 30 codons of the yeast *HIS4* gene to the *Escherichia coli lacZ* gene, which encodes β-galactosidase. In the plasmid pMB38-9merWT, a short linker connecting the two genes includes the Ty1 heptameric frameshifting site fused to *lacZ* in the +1 reading frame. In a second plasmid, pMB38-9merFF, a single nucleotide deletion in the heptamer places the *lacZ* gene in the 0 reading frame so its expression does not require frameshifting. The two plasmids are transformed separately into the recipient strain. Frameshifting efficiency is calculated as the ratio of expression from pMB38-9merWT to that of pMB38-9merFF.

### Polysome analysis

Sucrose gradient analysis of yeast ribosomes was performed essentially as described [34]. Briefly, 200 ml of each strain were grown in YPED medium to mid-exponential phase and harvested after addition of 10 mg cycloheximide. After washing, cells were lysed with glass beads and 40 A_260_ units of supernatant was layered on a 10 to 50 % sucrose gradient and centrifuged in an SW40 rotor for 4 h at 41,000 rpm. Fractions were collected and continuously analyzed for absorption at 260 nm using an ISCO Foxy Jr fraction collector.

### Northern analysis

The steady-state level of Ty1 mRNA was determined essentially as described [35]. Total cell RNA was isolated by the acid-phenol method [36] and 5 μg was separated by electrophoresis in 1 % agarose-glyoxal-DMSO gels and blotted to Brightstar-Plus positively charged nylon membranes (Life Sciences). For poly(A)^+^ RNA purification, total RNA was prepared using the MasturePure yeast RNA purification kit (Epicentre Biotechnologies, Madison, WI). Poly(A)^+^ RNA was isolated from 250 μg total RNA using the NucleoTrap mRNA purification kit (Clontech, Mountain View, CA). A DNA probe obtained as a 1.6 kb PvuII-ClaI fragment of the Ty1 *POL* gene and as a 1.4 kb EcoRI-XbaI fragment of the *PYK1* gene were labeled by random priming using α-[^32^P]dATP using the Deca Prime II kit (Life Sciences). In vitro transcription of Ty1 *GAG* (nt 1266-1601) was performed using a MAXIscript kit (Life Technologies, Carlsbad, CA) and α-[^32^P]UTP (3,000 Ci/mmol; Perkin Elmer, Waltham, MA). Hybridization was visualized by autoradiography or by image analysis using a STORM 840 phosphor imager (GE Healthcare). The experimental results shown in the figure are representative of three experiments performed.

### Western blot analyses

Three-milliliter SC-Ura liquid cultures were grown at 20 °C until saturated, which occurred between 24 and 48 h for different mutants. Strains were grown under similar conditions but split into different groups according to growth rate, and each group contained a wild type control. Total cell protein was prepared as previously described [37]. Protein isolated by trichloroacetic acid (TCA) extraction [38] from wild type and the *rpl1bΔ* mutant either expressing pGTy1 or not was also subjected to immunoblotting. Galactose-induction of cells containing pGTy1 was performed as previously described [6]. Protein concentration was determined using Coomassie Plus (Bradford) Assay Reagent (Thermo scientific, Waltham, MA). Protein samples were separated on a 10 % SDS-PAGE gel, and then transferred onto polyvinylidene difluoride (PVDF) membranes. Membranes were blocked in 5 % powdered milk–Tris buffered saline (100 mM Tris–HCl, 150 mM NaCl pH 7.5) with 0.1 % Tween 20 (TBST) and then incubated with primary antibody for 1 h at room temperature. Rabbit antisera directed against Ty1 VLPs (used to detect Gag; gift of Alan J. Kingsman), recombinant p18 (used to detect Gag and p22/p18) [21], and the control protein Hts1p (Gift of Thomas L. Mason) were used at 1:7,000, 1:5000 and 1:40,000 dilutions, respectively. Blots were washed three times for 10 min each in TBST. Primary antibody was detected with ECL anti-rabbit IgG, Horseradish peroxide linked whole antibody from donkey (GE healthcare, Pittsburgh, PA) at a 1:4,000 dilution in TBST for 1 h. Blots were washed three times for 10 min each in TBST, visualized by ECL Western Blotting Detection Reagents (GE healthcare, Pittsburgh, PA) and exposed to X-ray film. The experimental results shown are representative of two or three experiments performed.

## Results

### Identifying Ty1 host cofactor and restriction genes involved in protein synthesis

We have previously described screens to identify Ty1 cellular cofactor [6] and restriction genes [5] using a Ty1 mobility assay. The assay employs a plasmid (pJC573) bearing a modified Ty1 element, Ty1*his3-AI* [33]. *HIS3* is inserted downstream of the *POL* gene opposite to the direction of Ty1 transcription and is transcribed from its own promoter; the gene is interrupted by the artificial intron (AI), which is oriented in the direction of Ty1 transcription. The Ty1 RNA expressed from this construct is spliced before undergoing reverse transcription, removing the disruption of the *HIS3* gene and resulting after its reintegration into the genome in complementation of the chromosomal *his3* deletion (His^+^). Most His^+^ cells result from transposition of the element. A minority of retromobility events can occur by homologous recombination with an endogenous Ty1 transposon [39].

Among 457 Ty1 cofactor genes isolated in various systematic screens of the viable deletion mutants [3–7], 71 encode ribosomal proteins, ribosome biogenesis factors and translation factors including 33 ribosomal proteins genes: *RPL1B*, *RPL4A*, *RPL6A*, *RPL7A*, *RPL14A*, *RPL15B*, *RPL16B*, *RPL18A*, *RPL19A*, *RPL19B*, *RPL20B*, *RPL21A*, *RPL21B*, *RPL27A*, *RPL31A*, *RPL33B*, *RPL34A*, *RPL37A*, *RPL39*, *RPL40A*, *RPL41B*, *RPL43A*, *RPP1A*, *RPP2B*, *RPS0B*, *RPS9B*, *RPS10A*, *RPS11A*, *RPS19A*, *RPS19B*, *RPS25A*, *RPS27B* and *RPS30A*. On the other hand, of the 91 identified Ty1 restriction genes only three are translation related [3, 5]. *ASC1* is an integral ribosomal protein of the 40S ribosomal subunit and is the yeast homolog of the mammalian Receptor of Activated C Kinase 1 (RACK1) protein [40]. *ARC1* is a cofactor for aminoacyl-tRNA synthetases [41] and *TRM7* encodes a tRNA 2′-O-ribose methyltransferase [42].

Quantitative assays of Ty1 mobility were performed as described [5] to assess the severity of the transposition defects caused by deleting 16 identified ribosome-associated Ty1 cofactor genes, eight encoding large subunit (60S) subunit proteins, five encoding small subunit (40S) proteins, two encoding biogenesis proteins of the 60S (*rrp6* [43]) or 40S (*rrp8* [44, 45]) subunit and a karyopherin gene (*kap123* [46]) functioning in nuclear export of 60S subunits. These 19 strains were chosen for study based on their showing a strong mobility defect in the initial screen. Two control genes that reduce transposition by a mechanism not known to be associated with translation were also tested: *BEM4*, involved in budding, cell polarity and in maintenance of telomere length [47], and *SPE3*, encoding spermidine synthase [48]. The assay employs a Ty1-*his3AI* transposon integrated upstream of the *HIS4* gene. As shown in Table 1, the frequency of transposition was significantly reduced for all of the twenty deletion mutant strains. For 16 ribosome-associated cofactor mutant strains the frequency of transposition averaged 4.0 × 10^−7^ or 10-fold lower than the wild type frequency of 4.0 × 10^−6^. The frequencies varied from a minimum of 1.9 × 10^−8^ (for *rpl39*∆) to a maximum of 8.1 × 10^−7^ (for *rpl41B*∆). The mobility of the two control strains was also much less than wild type. For two mutant strains, *kap123*∆ and *bem4*∆, we were unable to observe any mobility events and so can only estimate a upper bound for the mobility frequency that is at least 73 and 340-fold below wild type, respectively. This secondary screen validates the identification of these genes as Ty1 cofactor genes.

**Table 1:** Quantitative Ty1 mobility is reduced in ribosomal protein gene Ty1 cofactor mutants

Strain	Function^a^	Ty1 mobility ± SEM (× 10^−7^)^b^	Fold reduced from WT
WT	–	40 ± 2.0	–
*rpl1B∆*	LSU protein	4.7 ± 1.1	8.5
*rpl4A∆*	“	1.9 ± 0.56	21
*rpl15B∆*	“	2.6 ± 0.78	16
*rpl21A∆*	“	4.9 ± 1.3	8.1
*rpl27A∆*	“	6.5 ± 1.6	6.2
*rpl39∆*	“	0.19 ± 0.14	210
*rpl41B∆*	“	8.1 ± 1.9	4.9
*rpp2B∆*	“	3.0 ± 1.0	13
*rps0B∆*	SSU protein	5.6 ± 0.81	7.2
*rps9B∆*	“	4.7 ± 0.79	8.5
*rps10A∆*	“	4.8 ± 0.66	8.3
*rps19B∆*	“	1.4 ± 0.27	29
*rps25A∆*	“	7.8 ± 1.4	5.1
*rrp6∆*	LSU processing	2.8 ± 0.56	14
*rrp8∆*	SSU processing	4.0 ± 0.79	10
*kap123∆*	60S nuclear export	<0.54^c^	>74
*bem4∆*	Budding	<0.11^c^	>340
*spe3∆*	Polyamine synthesis	0.67 ± 0.67	59

^a^LSU = large (60S) ribosomal subunit; SSU = small (40S) ribosomal subunit

^b^Mobility was calculated from a minimum of five repeated experiments as number of His^+^ cells per number of total viable cells

^c^No observed mobility; the maximum mobility is less than assuming mobility calculated if one event had occurred in the number of assays performed

### Most translation associated Ty1 cofactor mutants impair ribosome biogenesis or function

Deficits in ribosomal proteins generally results in impaired ribosome biogenesis (reviewed in [49]). These defects include blocks to ribosome biogenesis events including rRNA processing, binding of other ribosomal proteins to the pre-ribosome and transport to the cytoplasm. We therefore expected that the translation-associated Ty1 cofactor mutants would show effects on ribosome biogenesis. Many of the mutants showed slowed growth rates, consistent with reduced ribosome availability, however since most ribosomal protein gene deletion mutants grow at a normal rate [50] this is a poor test of their effect on biogenesis. Therefore, we directly assessed the effect of a subset of the cofactor mutants by analyzing polysomes from the wild type control and 11 of the translation-associated cofactor gene deletions using sucrose density centrifugation [51].

**Fig. 1:**
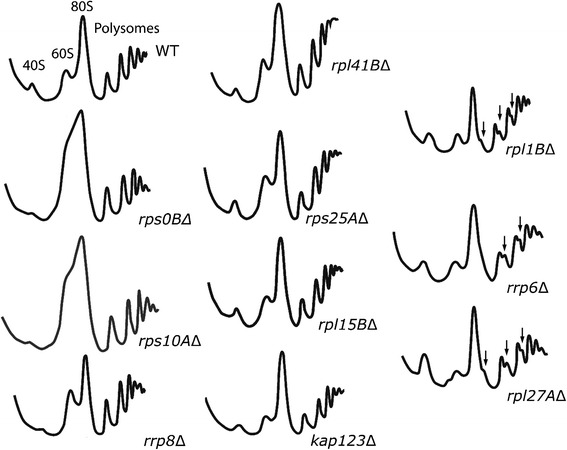
Most translation-associated cofactor mutants show reductions in the relevant ribosomal subunit. Sucrose gradient analyses for wild type (WT) and mutant strains. The Y-axis represents the absorbance at 260 nm (A_260_), proportional to RNA concentration, and the X-axis denotes increasing sucrose concentration with the lightest species eluting first (7–50 % sucrose). The 40S, 60S subunits, 80S monosome and polysomes in each of the profile are labeled and the presence of halfmers is denoted (black arrowhead)

Most of the mutants tested had obvious defects in subunit abundance. Mutants of 40S ribosomal proteins genes (*RPS0B* or *RPS10A*) or a 40S subunit processing factor gene (*RRP8*) were severely impaired in 40S assembly (Fig. 1). Each had 40S/60S ratios less than one-tenth of the wild type reflecting the near absence of free 40S subunits and increased amounts of 60S. The lack of Rrp8 was previously shown to cause reduced accumulation of mature 18S rRNA of the 40S subunit [44].

Similarly, lack of most large subunit protein genes tested showed evidence of reduced 60S accumulation or activity. Deletions of 60S ribosomal protein genes (*RPL1B* and *RPL27A*) or a 60S subunit assembly factor gene involved in 3′-end processing of 5.8S rRNA (*RRP6*) all resulted in reduced amounts of 60S subunits and all three showed evidence of “halfmers”, which are secondary peaks indicating complexes with masses slightly greater than a 70S or polysome peak. Halfmers are caused by the presence of mRNA-bound 43S pre-initiation complexes to which 60S subunits have failed to assemble in addition to one or more 80S ribosomes on an mRNA [52]. These peaks are direct evidence of slowed 60S subunit recruitment.

Four mutants displayed profiles resembling the wild type; the deletion of these genes does not appear to grossly alter the rate of assembly of either subunit. These genes encode a 40S ribosomal protein (*RPS25A*) two 60S proteins (*RPL15B*, *RPL41B*) and a ribosome nuclear export factor (*KAP123*). Previous work showed that lack of the 60S Ty1 cofactor gene *RPP2B* also does not alter subunit abundance [53]. Of the five encoded proteins, only Rpl15 is essential for viability; the proteins encoded by the other four can be eliminated without affecting viability although only a strain lacking Rpl41 grows at a normal rate [50]. These five proteins must affect Ty1 mobility without altering ribosome biogenesis.

### Most ribosomal protein gene deletions cause a significant reduction in Ty1 frameshift activity

An obvious reason for the connection between translation and Ty1 retrotransposition could be the Ty1 + 1 programmed frameshifting event responsible for expression of the Gag-Pol fusion protein. The stoichiometry of Gag to Gag-Pol sensitively controls transposition efficiency and even slight changes in the ratio of Gag to Gag-Pol proteins can block retrotransposition [12]. For Ty1, increasing frameshifting blocks transposition by causing incomplete proteolytic processing of the Gag-Pol polyprotein leading to formation of defective VLPs [14]. Reducing Ty1 frameshifting also blocks transposition [17] although the mechanism of this blockage is not known.

Frameshift activity in mutant strains was determined using a well characterized β-galactosidase reporter construct [13]. The construct has the first 33 codons of the *HIS4* gene fused to the β-galactosidase gene through a minimal Ty1 frameshift site with expression of the enzyme requiring frameshifting. The percent frameshift activity is expressed as the ratio of the frameshift activity to that of a frame fusion control in which the genes are in one open reading frame so expression does not require frameshifting. The use of the frame fusion control eliminates other transcriptional, post-transcriptional and translational effects on the activity of the enzyme.

**Fig. 2:**
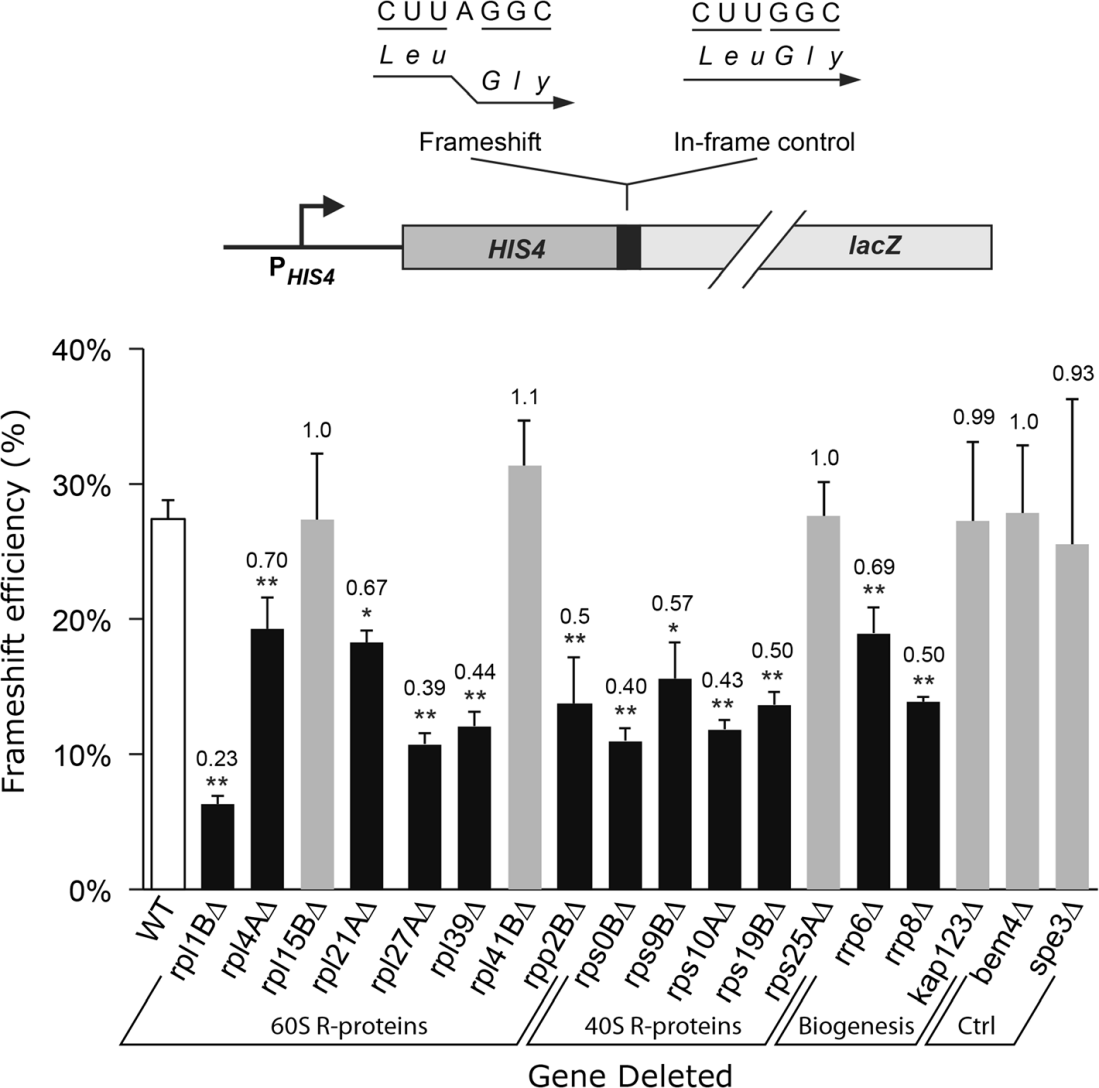
Analysis of translation-associated cofactor mutants for Ty1 + 1 programmed frameshift efficiency. The frameshift activity was measured using a β-galactosidase construct. The percent frameshift efficiency is reported as the activity of the frameshift construct relative to the frame fusion control construct. The unshaded column represents the frameshift activity for the wild type (WT) strain BY4741. The assays were repeated at least three times, each assay performed in triplicate. Asterisks indicate the frameshift activities of deletion strains that were significantly different from wild type activity as measured using ANOVA followed by the Tukey’s test (one asterisk, *P* ≤ 0.05; two asterisks *P* ≤ 0.005). Error bars represent the SEM. The ratio of the frameshift efficiency to the wild type appears above each column

Figure 2 shows the frameshift efficiency of the wild type (white column) and the same 18 Ty1 cofactor deletion strains tested in Table 1. We found that 12 of the mutant strains showed significantly lower frameshifting efficiency than the wild type (*P* < 0.05 or 0.005). These included five small subunit genes, six large subunit genes and two ribosome biogenesis genes. These deletions each had decreased frameshift efficiencies that averaged 2.2-fold lower than wild type and varied from 1.5 to 4.5-fold. The deletion mutants not known to be involved in ribosome function or assembly were included as controls: *SPE3* and *BEM4* had no significant effect on frameshift efficiency. Four translation-associated cofactor deletion mutants (*rps25A*∆*, rpl15B*∆, *rpl41B*∆ and *kap123*∆) also had frameshift efficiencies that were not significantly different from wild type; these mutants were the four that also showed near normal polysome profiles. The complete correspondence between these two phenotypes suggests a mechanistic connection between reduced ribosome biogenesis and reduced programmed frameshifting. The reduction in frameshift efficiency in about half of the tested mutants is 2-fold or more. Similar changes in stoichiometry blocked replication in the L-A virus [18] suggesting that this change in stoichiometry could significantly reduce transposition. Several of the mutants showed a less than 2-fold decrease (*rpl4A*∆, *rpl21A*∆ and *rrp6*∆) and the four mentioned above showed no decrease at all. We conclude that reduced frameshift efficiency probably contributes to the Ty1 mobility defect in most of the mutants but the full decrease in mobility may result from other abnormalities.

Comparison of the frameshift and transposition phenotypes of each of the mutants showed no significant correlation (Pearson’s correlation coefficient, *r* = 0.07). If we exclude the mutants that had no effect on frameshifting or the polysome profile the correlation is better (*r* = 0.27) but still weak. This statistical analysis suggests that the magnitude of the effect on transposition does not correlate well with the magnitude of the reduction of frameshifting, suggesting that effects beyond frameshift efficiency explain the reduction in Ty1 mobility. Clearly, for translation-associated cofactor mutants that do not alter either frameshifting or the polysome profile the effect on transposition must be from another cause, possibly extraribosomal [54].

### Post-transcriptional regulation reduces Ty1 Gag protein accumulation in most Ty1 translation-associated cofactor mutants

**Fig. 3:**
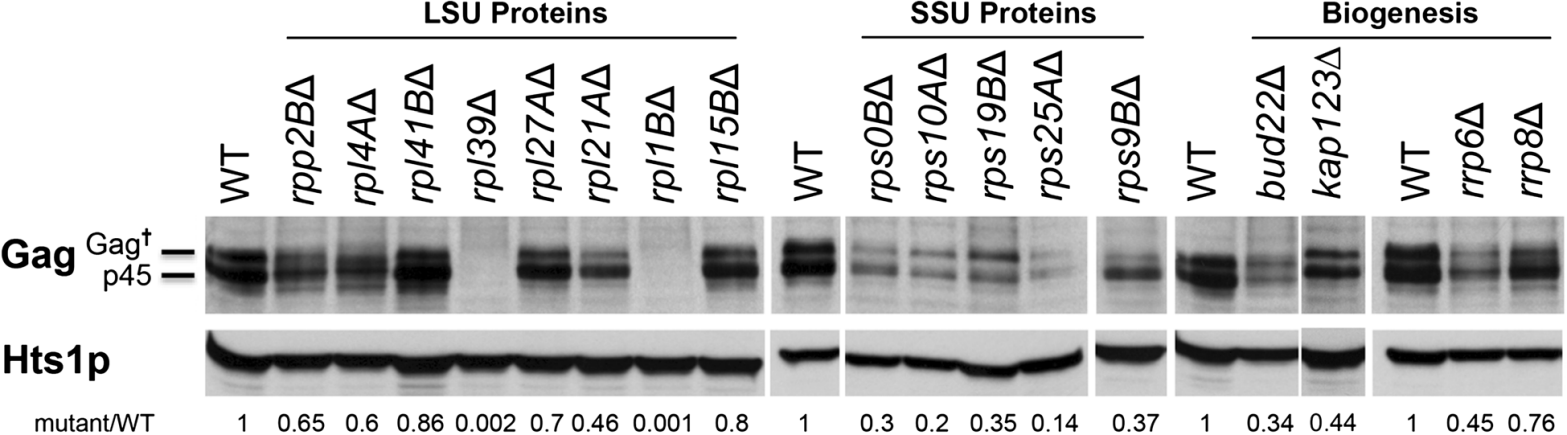
Ty1 Gag protein expression in various mutants defective in ribosomal biogenesis. Total cell protein was extracted from wild type (WT) and mutant strains, and subjected to Western blot analysis (50 μg/lane) using anti-VLP (upper panel) and control Hts1p (histidyl-tRNA synthetase; lower panel) antibodies. The Gag precursor (p49) and altered forms of Gag (Gag^†^), which co-migrate under these electrophoretic conditions, and mature (p45) protein are indicated. Hts1p was used as a loading control. The extent of antibody reaction to both p49/Gag^†^ and p45 species of Gag, quantified using ImageJ, is shown beneath each cofactor mutant strain expressed as a ratio to that of the wild type strain. LSU = large (60S) ribosomal subunit; SSU = small (40S) ribosomal subunit

Because most translation-associated Ty1 cofactor genes reduce 80S availability and programmed translational frameshifting, we suspected that they might also affect translation efficiency, in particular of the Ty1 Gag protein. The steady state level of Ty1 Gag, therefore, was determined for the wild type and 15 of the mutants tested in Table 1 by Western blotting using an anti-VLP antibody that strongly reacts with Gag [55]. Based on expression studies with cells expressing a Ty1 on a *GAL1*-promoted pGTy1 plasmid, we expected to see both the primary translational product, Gag-p49 and the mature, C-terminally processed form, Gag-p45 [56]. However, recent work suggests that alternate forms of endogenous Gag in addition to p45 are detected in normal cells [22]. As shown in Fig. 3, the wild type consistently showed approximately a 2-fold excess of endogenous p45 over the slower migrating bands that contain altered forms of Gag and perhaps p49 (denoted as Gag^†^). Surprisingly, we were unable to detect either endogenous Gag protein in the *rpl1B*∆ and *rpl39*∆ cofactor mutants. The lack of Gag might predict an extremely severe Ty1 mobility deficit and the *rpl39∆* mutant does have the lowest frequency of mobility of the deletion mutants tested, 210-fold lower than wild type and 25-fold lower than the average of the other mutants (Table 1). By contrast, the mobility frequency of the *rpl1B*∆ mutant was near the average of all mutants tested. To determine if Gag might be insoluble in the *rpl1B*∆ mutant, total protein from wild type and the mutant was prepared by TCA extraction and immunoblotted. Even under these harsher extraction conditions, which were developed to monitor transport of proteins into mitochondria [57], endogenous Gag was not detected in *rpl1B*∆ (Additional file 1: Figure S1). However, Gag was detected if Ty1 was overexpressed from pGTy1 and, even though Gag was mostly present in the altered form in the mutant, Ty1 mobility was restored to almost wild type levels. Similar results were also obtained with an antipeptide Gag antibody (data not shown). It is unclear why *rpl1B*∆ does not show a more severe phenotype given the severity of the putative Gag accumulation deficit. Perhaps *rpl1B*∆ Gag is present in a modified form that fails to enter the gel, is poorly electroblotted, or no longer reacts with Gag antisera.

Previously, we reported that the 40S assembly factor mutant *bud22*∆ accumulated lower amounts of Gag and higher amounts of Gag-p49 or Gag^†^ compared to Gag-p45 [6]. We confirmed the *bud22*∆ phenotype (Fig. 3) and found that three of the mutants tested here showed a similar phenotype (*rps10A*∆, *rps19B*∆ and *rps25A*). Three other mutants, *rps0B*∆, *rps9B*∆ and *rrp6*, accumulated similarly reduced amounts of Gag but do not show reduced processing of Gag-p49 to Gag-p45. All of these five genes encode 40S ribosomal proteins or 40S biogenesis proteins implying a link between 40S availability and the Bud22 phenotype and suggesting that the phenotype results specifically from a reduction in 40S availability.

The phenotypes of the remaining 60S ribosomal protein mutants are quite different than those of the 40S mutants. First, none of the 60S ribosomal protein mutants show an obvious deficit in p45 processing; the amount of the processed Gag-p45 is consistently greater than that of unprocessed Gag-p49/Gag^†^. Second, the 60S mutants, other than *rpl1B∆* and *rpl39*, clearly accumulate higher amounts of Gag than the 40S mutants although most express less than wild type (>50 % of wild type level). The co-occurrence of these two effects suggests that Gag processing or other posttranslational modifications [22] may explicitly depend on a sufficient supply of Gag protein. Reduced numbers of Gag proteins relative to Ty1 genomic RNA in the 40S mutants could result in increased production of defective VLPs. Combined with a reduction in the frequency of programmed translational frameshifting in these mutants, which reduces the ratio of Gag to Gag-Pol to far less than 50:1, many of these VLPs might then actually lack the protease activity of Ty1 Pol protein, blocking processing of the Gag and presumably Pol proteins as well. For the 60S mutants, the increased amounts of Gag and Gag-Pol per genomic RNA might ensure the presence of protease in more of the VLPs.

### The steady state level of full-length Ty1 mRNA cannot explain observed differences in Gag accumulation

**Fig. 4:**
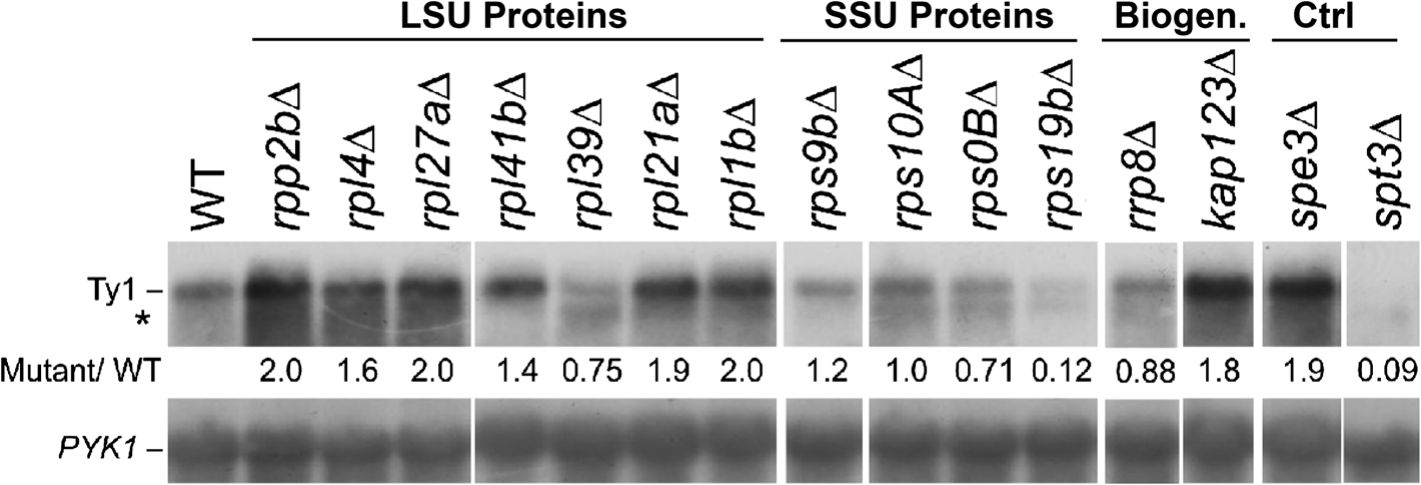
Subunit-specific changes in the steady-state level of Ty1 mRNA by cofactor mutants. Total cellular RNA was hybridized with an [^32^P]-labeled DNA probe specific to the mRNA encoding the RT domain of the Ty1 element (upper panel) or *PYK1* control transcript (lower panel), which serves as a lane to lane control for the amount of RNA in the samples. The intensity of hybridization by the Ty1 probe relative to the *PYK1* probe was quantified using the ImageJ software. The asterick (*) shows the position of the internally initiated Ty1i RNA detected in some of the mutants. LSU = large (60S) ribosomal subunit; SSU = small (40S) ribosomal subunit

The Ty1 promoter is complex and extends over the first approximately 1000 bp of the element, including sites upstream of the start site of transcription and downstream within the *GAG* gene [58]. The region includes binding sites for six transcription factors: Gcn4, Gcr1, Mot3, Ste12/Tec1, Mcm1, Tea1 and Rap1. Since Gcn4 and Rap1/Gcr1 regulate ribosomal protein genes [59, 60], it was reasonable to suspect that deficits in ribosomal protein accumulation might indirectly affect transcriptional regulation of chromosomal Ty1 elements. To test this idea, total RNA was isolated from ribosomal protein deletion strains grown at 20°C, a temperature conducive to Ty1 transposition [61] and was hybridized with radiolabeled probes specific to Ty1 (RT domain) and *PYK1*, which served as a normalization control (Fig. 4). The Spt3 protein, a component of SAGA complex [25], is required for chromosomal Ty1 element transcription [62]. Although these strains contained a plasmid-borne copy of Ty1*his3-AI* integrated near *HIS4* [5], the level of Ty1*his3-AI* RNA was not easily detected due to the much higher amount of total Ty1 RNA, which is similar to earlier results obtained with chromosomal Ty1*his3-AI* elements [33]. As expected, endogenous Ty1 transcript production is severely reduced in an *spt3*Δ strain, providing the negative control in this experiment. The steady state amount of Ty1 transcript in the 60S subunit mutants was about 2-fold greater than wild type. An exception is *rpl39*∆, which accumulates about 75 % of wild type. Mutants affecting 40S subunit proteins, by contrast, accumulated slightly reduced amounts of Ty1 mRNA corresponding to 75–100 % of wild type. In general, the Ty1 mRNA levels of these mutants correlated poorly with the amount of Ty1 Gag produced from each. As shown in Fig. 3, none of the 60S subunit protein mutants expressed increased Gag (especially true for the two mutants with severely reduced Gag accumulation, *rpl1B*∆ and *rpl39*∆) and the 40S mutants accumulated far less than predicted by mRNA accumulation. The lack of correlation indicates that the reduction in Gag accumulation is post-transcriptional, however, the mechanism of this effect remains to be determined. The simplest model is that the proportion of Ty1 mRNA in the translated pool may be reduced in the mutants, possibly because of reduced availability of 40S or 60S subunits.

### Ty1i RNA increases relative to Ty1 mRNA in several ribosomal subunit mutants

**Fig. 5:**
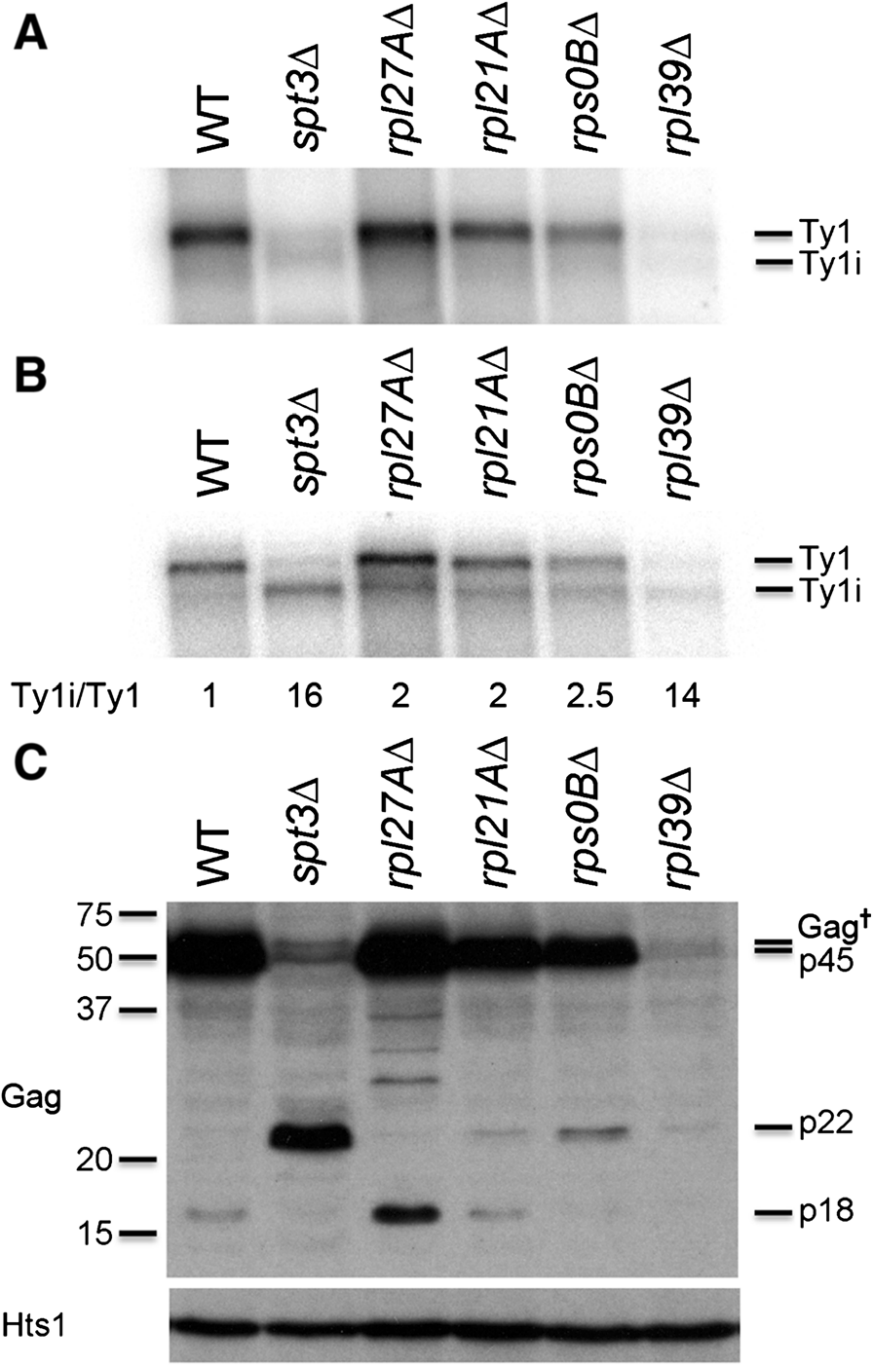
Ty1i RNA and Gag-p22/p18 expressed in ribosomal protein deletions. **a** Northern blot of total RNA from wild type (WT) and four mutant strains (*spt3∆, rpl27A∆, rpl21A∆, rps0B∆* and *rpl39*). A ^32^P-labeled riboprobe of Ty1 *GAG* (nt 1266-1601) hybridized to with full-length Ty1 mRNA (“Ty1”) and, especially in the *spt3∆* mutant strain, to the subgenomic inhibitory Ty1i mRNA (“Ty1i”). **b** A Northern blot of poly(A)^+^ mRNA from the same strains. The same probe hybridized to Ty1 and Ty1i mRNAs. **c** Total protein extracts from the same strains was immunoblotted with a p18-specific antiserum to detect p49/p45/Gag^†^ and p22/p18. The histidyl-tRNA synthetase (Hts1) served as a loading control

A subgenomic RNA (Fig. 4, denoted by the asterisk) was detected in several mutants, including *rpl27A*∆, *rpl21A*∆, *rps0B*∆, and *rpl39*∆, that may correspond to the newly discovered Ty1i transcript involved in controlling Ty1 copy number [21]. Therefore, we repeated the Northern analyses (Fig. 5) for wild type, *spt3*∆, and selected ribosomal subunit mutants using total (Fig. 5a) and poly(A)^+^ (Fig. 5b) RNA and a ^32^P-labeled riboprobe derived from Ty1 *GAG* (nt 1266-1601). As reported previously [21], wild type cells contain a low level of the 4.9-kb Ty1i transcript, which increases in abundance in an *spt3*∆ mutant. Interestingly, the level of Ty1i RNA increased in the four ribosomal subunit mutants tested. Hybridization signals were more evident in poly(A)^+^ RNA when compared with total RNA, perhaps because the relative amounts of polyadenylated Ty1 mRNA and Ty1i RNA differs [23]. Since recent results suggest that the relative levels of Ty1i and Ty1 mRNA are effective readouts for inhibition of Ty1 transposition by the Ty1i encoded product p22 [21], we compared the relative levels of Ty1i RNA and Ty1 mRNA. The *spt3*Δ mutation greatly increased the Ty1i/Ty1 RNA ratio since Spt3p is required for Ty1 mRNA but not Ty1i RNA transcription. The Ty1i/Ty1 transcript ratio also increased to a lesser extent in the ribosomal subunit mutants.

Ty1i mRNA is translated to produce an N-terminally truncated Gag-p22, which is likely the primary translational product, and a C-terminally processed Gag-p18 protein; the presence of these proteins leads to defective VLP assembly and function, and reduced Ty1 mobility [21]. We performed immunoblotting with an antibody raised against recombinant p18 on the same set of wild type and mutant strains and found evidence for p22/p18 in all of the mutants (Fig. 5c). As expected, the *spt3*∆ mutant accumulated large amounts of p22; because little Ty1-encoded proteins are expressed in this strain, no p18 was detected. In the four Ty1 ribosome-associated mutants there was evidence of p22 and/or p18. The amount of p18 was correlated with the amount of full-length Gag protein, which indicated the extent of translation of full-length Ty1 proteins. The *rpl27A*∆ mutant had both the highest amount of both Gag-p49/p45/Gag^†^ and Gag-p22. The increasingly lower amounts of Gag-p49/45 in the *rpl21A*∆, *rps0B*∆ and *rps39*∆ mutants correlated with increasingly lower amounts of Gag-p18 and an increased ratio of Gag-p22 to Gag-p18. These results confirm that the mutants tested express substantial amounts of Ty1i mRNA and the inhibitory Gag-p22/p18 proteins and suggest that the reduced Ty1 mobility in these strains in part results from this inhibitory mechanism.

## Discussion

The number and diversity of genes identified as host factors for Ty1 retrotransposition reflects the complexity of the Ty1 lifecycle [2]. Many host restriction and cofactor genes encode proteins involved in basic processes of cellular information transfer with those involved in protein synthesis being significantly overrepresented among the Ty1 cofactor genes, which are required for optimal level of transposition [7]. Similarly, mutations targeting 60S ribosomal proteins are also required for propagation of the yeast L-A double-stranded RNA virus [9]; L-A shares several features with retroviruses and retrotransposons (reviewed in [11]) so this shared mode of control may reflect similar mechanisms of propagation. Quantitative assays of Ty1 mobility (Table 1) validate the requirement for 13 ribosomal proteins genes and three ribosome biogenesis genes (*RRP6, RRP8* and *KAP123*). As expected, most but not all mutants lacking 40S or 60S structural proteins or biogenesis factors are deficient in the corresponding subunit. It has long been recognized that biogenesis of the two subunits diverges early in biogenesis with a 90S pre-ribosome containing immature forms of both subunits dividing into a pre-40S and pre-60S complexes (reviewed in [63]). No comprehensive test of the effect of ribosomal protein depletion on ribosome biogenesis has been performed but most of the proteins have been tested and in all cases the lack of a ribosomal protein blocks maturation and accumulation of the corresponding subunit [49, 64].

**Fig. 6:**
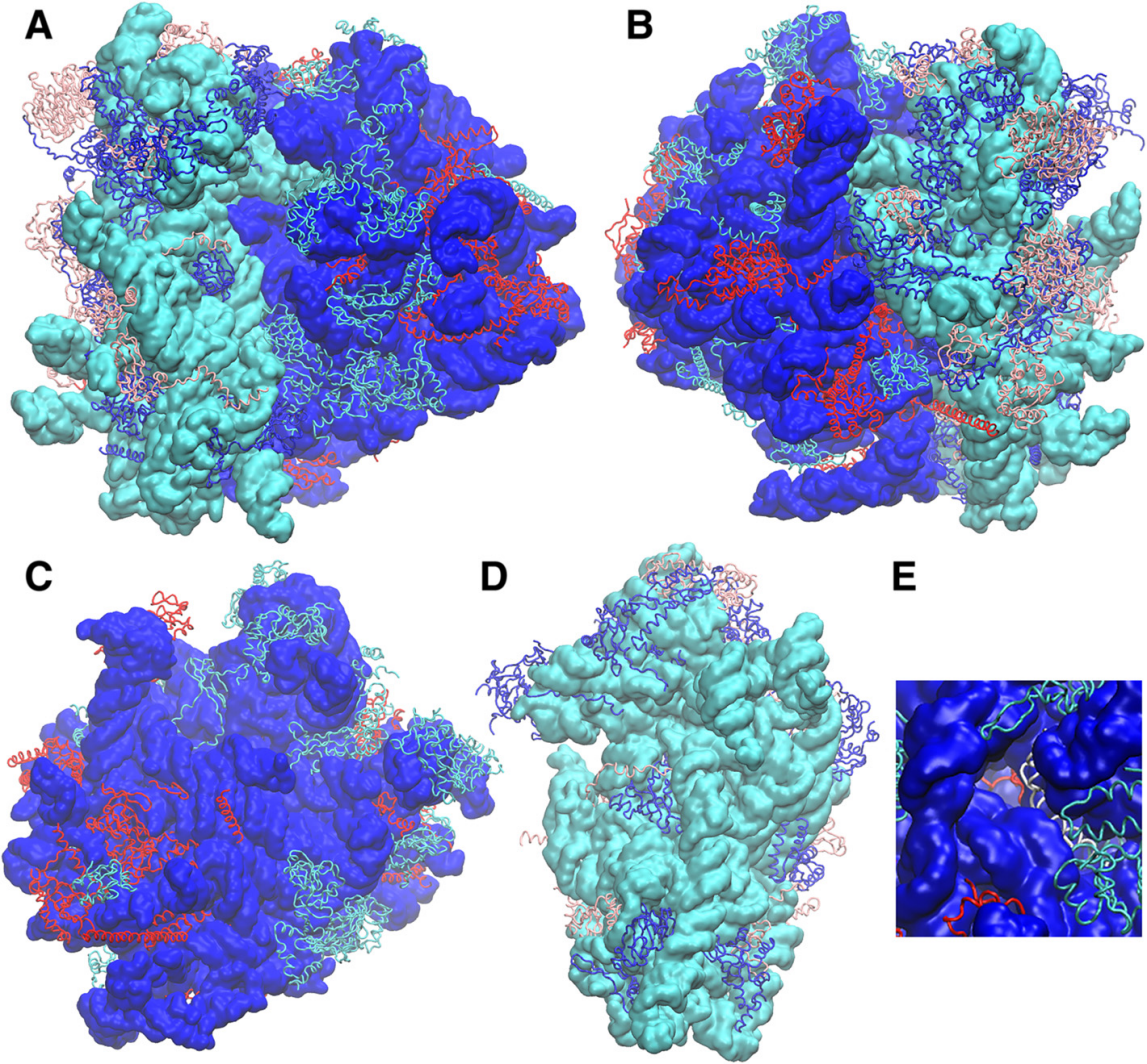
Distribution of Ty1 cofactor ribosomal proteins on the structure of the *S. cerevisiae* ribosome. The structure of the yeast ribosome [69] is derived from structure files 3J78 deposited in the Protein Data Bank (PDB; http://www.rcsb.org/pdb/) [70]. The structure was modeled using the VMD Molecular Graphics Viewer (http://www.ks.uiuc.edu/Research/vmd/) [71]. The structure of the rRNAs are shown as a surface with the large subunit rRNAs colored blue and the small subunit rRNA colored cyan. The ribosomal proteins are shown in cartoon mode with those encoded by Ty1 cofactor genes in red (large subunit) or pink (small subunit) and all others in cyan (large subunit) or blue (small subunit). **a** The 80S subunit seen from A site side. **b** The 80S subunit rotated 180° to view from the E site side. **c** The 60S subunit showing the surface that is in contact with the 40S in the 80S complex. **d** The 40S subunit showing the 60S interface surface. **e** The end of the nascent peptide channel from the peptide exits showing Rps39 (*in white*) located immediately inside the end on the right side of the exit channel; beyond Rps39, deep within the exit channel, the tip of a loop on Rps4 can be seen (*in red*)

The role of individual ribosomal proteins in ribosome biogenesis appears to be regional with proteins that bind in similar locations on the ribosome having roles in early, middle or late subunit biogenesis [49, 64]. Paradoxically, only a subset of ribosomal protein genes has been identified as Ty1 cofactors, totaling 33 of the 138 ribosomal protein genes. If this subset were a discrete group based on their function in ribosome function or biogenesis their protein products would be expected to cluster in a similar fashion in the ribosome. We have tested the Ty1 mobility phenotype of other ribosomal protein genes not previously characterized as Ty1 cofactor genes, 19 using the qualitative test and 12 of those using the quantitative test and found that each had reduced Ty1 mobility (Additional file 2: Table S1), expanding the number of ribosomal protein cofactor genes to 52 and suggesting strongly that most ribosomal proteins may in fact be encoded by cofactor genes that may have escaped detection because of differences in mutant strain backgrounds, transposition assays, or strength of the Ty1 mobility phenotype. These 52 genes encode 37 proteins, representing 47 % of the 80S proteins, are distributed throughout the structure of the 80S ribosome with no obvious evidence of clustering (Fig. 6a-d). This distribution strongly implies that their function in Ty1 mobility has little or nothing to do with their location on the ribosome, or any specific role in biogenesis or during translation. The Ty1 cofactor phenotype may be a generic effect of mutations that cause a significant reduction in subunit availability. A comprehensive analysis of the effect of ribosomal protein depletion on Ty1 mobility would confirm this conjecture but is outside the scope of this study.

Based on Gag protein expression, we can divide these genes into three groups: the 40S ribosomal protein genes (strongly reduced Gag accumulation and processing), the majority of the 60S genes (slightly reduced Gag accumulation but normal processing) and *rpl1B*∆ and *rpl39* (complete loss of detectable Gag; see Table 2 for a summary of all phenotypes). The reduction in accumulation of Gag is likely not transcriptional because of the lack of correlation between the accumulation of Ty1 mRNA and Gag protein. The effect could result from decreased protein stability or aberrant ER translocation [22] as we suspect in one case noted below, but given the primary defect is in availability of ribosome subunits, the most likely model is that translational insufficiency reduces Gag accumulation. The distinct effect on accumulation in the 40S and most 60S mutants could reflect a difference in the way that mRNAs compete for the two subunits. Binding of 40S subunits to individual mRNAs can differ widely among cellular transcripts based on sequence and structure with some mRNAs competing much more efficiently than others (reviewed in [65]). Recruitment of the 60S subunit should be less context dependent since the 60S mainly recognizes the 40S subunit once the initiation factors making up the 43S preinitiation complex have dissociated; there is no reason to suppose that some mRNAs compete more effectively at that stage of initiation. The greater reduction caused by reduced availability of 40S subunits, then, suggests that the Ty1 mRNA competes much less effectively for 43S preinitiation complex than does the average yeast mRNA. One reason for this could be that the Ty1 mRNA has an unusual structure and recent work has demonstrated that the 5′ end of the Ty1 mRNA forms a phylogenetically conserved RNA pseudoknot [66]. Mutational destabilization of the pseudoknot causes a modest increase in Gag accumulation, suggesting that the pseudoknot may inhibit Ty1 mRNA translation [66]. The same destabilizing mutations have the opposite effect—strongly decreasing Ty1 transposition—implying that the 5′ pseudoknot may play a structural role during the retrotransposition process [66]. These observations, however, do not contradict our finding that reducing 40S availability strongly reduces Gag accumulation. The highly structured nature of the 5′ end of the mRNA should reduce the efficiency of 43S complex binding, thus reducing the amount of Gag available to form virus-like particles. We do not imagine any direct effect of 40S availability on the role played by the 5′ pseudoknot during retrotransposition.

**Table 2:** Summary of experimental results

Gene	Function^a^	Ty1 moblility relative to WT	Frameshifting relative to WT	Polysome effects	Gag relative to WT	Ty1 mRNA relative to WT
*RPL1B*	LSU protein	0.12	0.23	↓60S/halfmers	0.001	2.0
*RPL4A*	“	0.48	0.70	↓60S/halfmers^b^	0.60	1.6
*RPL15B*	“	0.07	1.0	~WT	0.80	n.d.
*RPL21A*	“	0.12	0.67	n.d.	0.46	1.9
*RPL27A*	“	0.16	0.39	↓60S/halfmers	0.70	2.0
*RPL39*	“	0.005	0.44	↓60S^c^	0.002	0.75
*RPL41B*	“	0.20	1.1	~WT	0.86	1.4
*RPP2B*	“	0.08	0.50	WT^d^	0.65	2.0
*RPS0B*	SSU protein	0.14	0.40	↓40S	0.30	0.71
*RPS9B*	“	0.12	0.57	n.d.	0.37	1.2
*RPS10A*	“	0.12	0.43	↓40S	0.20	1.0
*RPS19B*	“	0.04	0.50	↓40S	0.35	0.12
*RPS25A*	“	0.20	1.0	~WT^e^	0.14	n.d.
*RRP6*	LSU processing	0.07	0.69	↓60S/halfmers	0.45	n.d.
*RRP8*	SSU processing	0.10	0.50	↓40S	0.76	0.88
*KAP123*	60S nuclear export	<0.01	0.99	~WT	0.44	1.8

^a^LSU = large (60S) ribosomal subunit; SSU = small (40S) ribosomal subunit

^b^Ohtake et al. [72] ^c^Sachs & Davis [73] ^d^Cardenas et al. [53] ^e^Léger-Silvestre et al. [74]

The phenotype of the *rpl1B*∆ and *rpl39*∆ mutants is quite distinctive. The lack of accumulation of Gag in these mutants despite the presence of Ty1 mRNA strongly suggests a post-transcriptional block. The striking inability to detect Gag protein in the *rpl39*∆ mutant is consistent with it having the lowest Ty1 mobility frequency of any of the mutants, 210-fold less than wild type. The *rpl1B*∆ mutant with a similar Gag accumulation phenotype, however, supports Ty1 mobility slightly more than the average of the mutants tested. Because the *rpl1B*∆ mutation causes a relatively small decrease in mobility we suspected that the low level of Gag detected in the *rpl1B*∆ mutant results from its being sequestered and not easily extracted. A harsher method of extraction detected no more Gag protein but overexpressing the Ty1 mRNA in this mutant background using a Gal-driven element resulted in significantly more Gag detected but also restored near normal Ty1 mobility. We cannot exclude that Gag is sequestered in the *rpl1B*∆ mutant and we are unable to explain why, despite their similar Gag phenotype, the *rpl1B*∆ and *rpl39* mobility phenotypes are so different. The location of the Rpl39 protein in the ribosomal subunit provides a clue to the origin of the difference. *RPL39* being a single copy gene, the deletion mutant accumulates 60S subunits lacking the protein. Rpl39 is located at the opening of the peptide exit tunnel (see Fig. 6e) and interacts with the hydrophobic signal anchor sequence of a nascent protein during co-translational insertion into the endoplasmic reticulum (ER) [67]; this interaction appears to be important for targeting proteins to the ER [68]. Doh et al. [22] demonstrated that VLP assembly sites are nucleated by targeting of ribosomes translating Ty1 mRNAs to the ER by contranslational insertion of Ty1 proteins into the ER. The formation of cytoplasmic foci [22], called T-bodies [23] or retrosomes [24] may be necessary for efficient formation of VLPs and therefore for maximal Ty1 mobility. Ty1 Gag is synthesized but is less stable when targeting to the ER is blocked. This suggests that a block to ER targeting caused by the absence of Rpl39 inhibits VLP assembly and Gag accumulation, resulting in a severe transposition defect.

## Conclusions

The overall conclusion of this work is that failure in ribosome biogenesis results in reduced Ty1 mobility with distinct phenotypes for mutants deficient in 40S and 60S subunit proteins. The effect shows no clear connection to a particular step in biogenesis or the position of the protein within either subunit. The effect is largely translational involving both decreased programmed translational frameshifting, reduced efficiency of translation and possibly increased instability of newly synthesized Ty1 Gag protein. A connection has been made to co-translational insertion of Ty1 Gag protein into the endoplasmic reticulum both by the severe phenotype of a mutant lacking the Rpl39 protein, which plays a role in targeting co-translational ER insertion and our demonstration of the accumulation of the Ty1i protein and Gag-p22/p18 in several of the translation-associated Ty1 cofactor mutant strains. Experiments are continuing to determine whether the connection between ribosome subunit sufficiency and Ty1 mobility is through the disruption of this newly discovered step in the Ty1 transposition process. Future studies will address how the individual pathways identified here modulate Ty1 gene expression and function, and whether similar processes also affect other retroelements.

## Additional files

Additional file 1: Figure S1. Ty1 Gag level and Ty1*his3-AI* mobility in an *rpl1B*Δ mutant. Total cell protein was prepared by trichloroacetic acid extraction form wild type and *rpl1B*Δ mutant cells that were induced for expression of pGTy1*his3-AI* or not. Ty1 Gag-p45 and slower migrating forms of Gag (†) were detected by immunoblotting with VLP antiserum. Histidyl tRNA synthetase (Hts1) served as a loading control. Relative Ty1*his3-AI* mobility from galactose-induced cells was determined by dividing the frequency of Ty1*his3-AI* mobility obtained in the *rpl1B*Δ mutant [6.4 × 10^−4^ (0.6)] by the wild type [9 × 10^−4^ (0.9)] as described previously [21]. (TIFF 813 kb) Additional file 2: Table S1. Identification of novel Ty1 mobility genes. Given the over representation of ribosomal protein gene deletions among the identified Ty1 mobility genes, we extended screen to include  ribosomal protein genes that had not previously been identified in any large scale screen. Each of 19 additional genes were screened first qualitatively with all showing defects in transposition as shown. The mobility phenotypes of most of these mutants were then quantitated as shown. (DOC 3310 kb)

## Abbreviations

ER: endoplasmic reticulum
Gag: group specific antigen
L-A: a Large double-stranded RNA virus of *Saccharomyces cerevisiae*
LSU: large ribosomal subunit
LTR: long terminal repeat
POL: POLymerase; the retroviral polyprotein processed to produce the protease, reverse transcriptase, RNase H and integrase enzymes
RT: reverse transcriptase
SSU: small ribosomal subunit
Ty: transposon of yeast
VLP: virus-like particle
