# Erratum to: ribosomal protein and biogenesis factors affect multiple steps during movement of the Saccharomyces cerevisiae Ty1 retrotransposon

**DOI:** 10.1186/s13100-016-0060-1

**Published:** 2016-02-09

**Authors:** Susmitha Suresh, Hyo Won Ahn, Kartikeya Joshi, Arun Dakshinamurthy, Arun Kannanganat, David J. Garfinkel, Philip J. Farabaugh

**Affiliations:** 1 Department of Biological Sciences and Program in Molecular and Cell Biology, University of Maryland Baltimore County, Baltimore, 21250 MD USA; 2 Department of Biochemistry & Molecular Biology, University of Georgia, Athens, 30602 GA USA; 3 Present address: Division of Infectious Diseases, Department of Internal Medicine, Stanford University School of Medicine, Stanford, 94305 California USA; 4 Present address: Department of Nanosciences and Technology, Karunya University, Karunya Nagar, Coimbatore, 641 114 Tamil Nadu India

## Erratum

Unfortunately, the original version of this article [1] contained an error. The author’s name, ‘Arun Kananganat’ , was spelt incorrectly. This can be found corrected in the author list above.
