# Supplementary figures and images for: Ribosomal protein and biogenesis factors affect multiple steps during movement of the Saccharomyces cerevisiae Ty1 retrotransposon
Source: Mob DNA. 2015 Dec 8;6:22. doi: 10.1186/s13100-015-0053-5 (PMC4673737; doi:10.1186/s13100-015-0053-5)

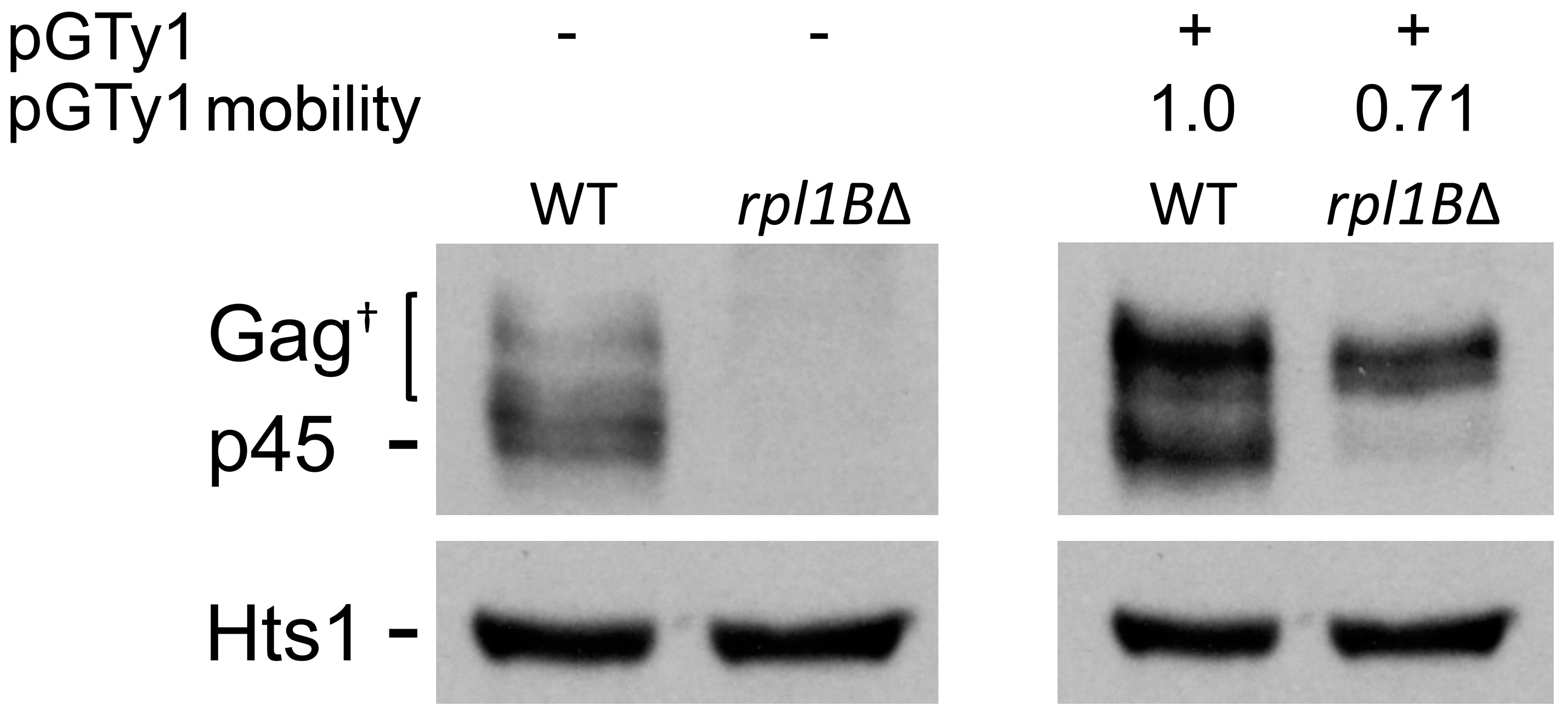

Supplement: Additional file 1: Figure S1. — Ty1 Gag level and Ty1his3-AI mobility in an rpl1BΔ mutant. Total cell protein was prepared by trichloroacetic acid extraction form wild type and rpl1BΔ mutant cells that were induced for expression of pGTy1his3-AI or not. Ty1 Gag-p45 and slower migrating forms of Gag (†) were detected by immunoblotting with VLP antiserum. Histidyl tRNA synthetase (Hts1) served as a loading control. Relative Ty1his3-AI mobility from galactose-induced cells was determined by dividing the frequency of Ty1his3-AI mobility obtained in the rpl1BΔ mutant [6.4 × 10^−4^ (0.6)] by the wild type [9 × 10^−4^ (0.9)] as described previously [21]. (TIFF 813 kb) [file 13100_2015_53_MOESM1_ESM.tiff]
