# Supplementary material for: Ribosomal protein and biogenesis factors affect multiple steps during movement of the Saccharomyces cerevisiae Ty1 retrotransposon
Source: Mob DNA. 2015 Dec 8;6:22. doi: 10.1186/s13100-015-0053-5 (PMC4673737; doi:10.1186/s13100-015-0053-5)
Supplement: Additional file 2: Table S1. — Identification of novel Ty1 mobility genes. Given the over representation of ribosomal protein gene deletions among the identified Ty1 mobility genes, we extended screen to include ribosomal protein genes that had not previously been identified in any large scale screen. Each of 19 additional genes were screened first qualitatively with all showing defects in transposition as shown. The mobility phenotypes of most of these mutants were then quantitated as shown. (DOC 3310 kb) [file 13100_2015_53_MOESM2_ESM.doc]

Supplemental Table 1

| Gene | Function^1^ | Qualitative mobility defect^2^ | Quantitative mobility (x 10^-7^) |
| --- | --- | --- | --- |
| Wild type | n.a. | + | 40 ± 2.0 |
| *RPL2A* | LSU protein | – – | 11 ± 3.4 |
| *RPL2B* | “ | – – | 7.0 ± 1.9 |
| *RPL4B* | “ | – – | <0.09 |
| *RPL8A* | “ | – | n.d. |
| *RPL8B* | “ | + / – | n.d. |
| *RPL22A* | “ | – – | 0.26 ± 0.15 |
| *RPL22B* | “ | – | 18 ± 3.9 |
| *RPL26B* | “ | – – | 1.8 ± 0.06 |
| *RPL40B* | “ | – – | <0.08 |
| *RPL41A* | “ | – | 8.8 ± 2.0 |
| *RPS0A* | SSU protein | – | 22 ± 2.3 |
| *RPS7A* | “ | – | n.d. |
| *RPS9A* | “ | – | 24 ± 3.7 |
| *RPS10B* | “ | + / – | 17 ± 1.9 |
| *RPS14A* | “ | – | n.d. |
| *RPS21A* | “ | – | n.d. |
| *RPS24A* | “ | – – | n.d. |
| *RPS24B* | “ | – – | 4.4 ± 0.9 |
| *RPS25B* | “ | – | n.d. |

^1^ LSU = large (60S) ribosomal subunit; SSU = small (40S) ribosomal subunit

^2^ “WT” = hundreds of papillae per sector; “+/–” = tens to hundred papillae per sector; “–” = ~ ten papillae per sector; “– –” = zero to several papillae per sector
